# Health policy under the microscope: a micro policy design perspective

**DOI:** 10.3389/fpubh.2023.1180836

**Published:** 2023-06-14

**Authors:** Giliberto Capano, Federico Toth

**Affiliations:** Department of Political and Social Sciences, University of Bologna, Bologna, Italy

**Keywords:** comparative health governance, policy design, policy instruments, regulation, expenditure, taxation, information

## Abstract

The comparative study of health policy has focused mainly on the macro-structural dimensions of health systems and reforms that have sought to change these organizational arrangements. Thus, a great deal of attention has been paid to the multiple models of insurance against sickness risks and various modes of organizing and financing healthcare providers. However, little attention has been paid to policy tools and policy design in the health policy domain. This research gap largely impedes a focus on the micro (granular) dimension of health policy, although this is the level at which health policies impact reality and thus deliver progress toward the expected goals. Such a focus on the micro dimension could not only allow a finer-grained comparison of how health systems work but also shed light on how capable health policies are of achieving the expected outcomes. This paper fills this gap by presenting an analytical framework capable of illuminating the granular dimension of policy design (the instrumental delivery package) and shows the analytical relevance of the framework by applying it to the designs of maximum waiting time guarantee and vaccination mandate policies.

## 1. Introduction

The national responses to the COVID-19 pandemic and the different strategies adopted by governments to promote vaccination campaigns have made it clear how crucial policy tools are. Often, it is not so much (or not only) the policy objectives that differentiate the strategies adopted in different countries but rather the policy instruments selected (and combined) to pursue the desired goal. Governments often have the same objective but try to achieve it with very different policy instruments or combinations thereof. The way the tools are designed and combined makes a great deal of difference: in practice, in regard to implementation, some instruments (or policy mixes) prove to be more effective than others. The experience of the recent pandemic has therefore confirmed that there is a need to move beyond the study of macro- and meso-level institutional arrangements and dynamics toward a finer-grained analysis of the processes of health policymaking. In other words, the literature on policy design and policy tools has thus far had little dialog with comparative health policy studies. This paper aims to begin to fill this gap by presenting a framework for analysing health policy instruments at a more granular level than that at which general categories of policy tools (such as regulation, public provision, information, and market incentives) are usually distinguished. We assume that these general categories of policy instruments cannot account for the concrete design of health policy and thus cannot completely shed light on the real mechanisms activated to reach the expected goal. The complexity of the dimensions of policy instruments has been highlighted in the health policy literature [see, for example (1)] but never conceptually developed.

Thus, in this paper, we attempt to present a conceptual treatment of policy instruments in health policy by adopting the theorizations proposed by the literature on policy design and policy instruments, following Salamon’s suggestion (2, 3) that the method of delivery of policy instruments is key. We propose a novel conceptualization of policy instruments with a specific focus on the components of the package designed to deliver each policy instrument. This conceptual treatment makes it possible to better understand how policies are steered and to assess their policy outcomes.

In the second section, the current state of the literature on the different dimensions of governance arrangements in health policy is summarized, and the weakness of this literature with respect to policy instruments is underlined. In the third section, a novel framework of analysis is presented. In the fourth section, the analytical value of the framework is illustrated by applying it to two different instruments that are widely used in the healthcare domain, namely, maximum waiting time guarantees and vaccination mandates. The fifth section is devoted to discussing the implications of the proposed framework. In the conclusion, some lines for further research are sketched.

## 2. Governance arrangements in health policy: the lack of a systematic perspective on policy instruments

### 2.1. The macro and meso perspectives of comparative health policy

To date, health policy scholars have focused mainly on the institutional structure of national health systems and on governance arrangements—in other words, on the macro and meso dimensions of this policy field. Systematic institutional comparative analyses have produced various typologies of health systems that also serve as typologies of how health policies are designed at the macro level. There are two different streams of classification in the field. One focuses on the main activities to be carried out and on the role played by the state (4–8), while the other is centered on how healthcare systems work, what services they provide and how patients access necessary healthcare services (9–12). The categories of healthcare systems arising from these typologies indicate—albeit not in a very detailed or convincing manner—how healthcare services are delivered. Overall, the most long-standing and densest typological traditions in health policy represent significant efforts to conceptualize and analyse governance arrangements at both the macro level (the first type of typology) and the meso level (the second type of typology). However, it is not clear whether there is congruence between the macro and meso levels of governance systems (13) and whether and how governance arrangements (at the various levels) truly matter in terms of health performance.

The focus on governance arrangements and dynamics in health systems has produced many theoretical frameworks explicitly conceptualized to better account for the characteristics and modes of operation of governance in healthcare. Many of these frameworks have been based on institutional and principal–agent theories that assume that governance in health policy is substantially driven by incentives or sanctions that are used to push agents toward the expected behavior and thus the planned performance and by information asymmetry and differential power among different stakeholders (14, 15). These frameworks emphasize that accountability is the leading governance mechanism in health policy. Another relevant theoretical stream is economic neo-institutionalism *à la* Douglas North (16–18), which postulates that health policy is the set of informal and formal rules according to which actors deal with opportunity constraints. These approaches consider governance in health policy as a multi-layered arrangement at the national and local levels and pay particular attention to the rules of the two strategic stages of the policy process: formulation and implementation.

### 2.2. Policy instruments in the healthcare sector: a still-undeveloped perspective

The above summarized literature on institutions, arrangements and governance in the health domain is very useful for describing health policies’ general mode of operation, but it is very ambiguous with respect to the real channels through which policies and systems impact the reality of healthcare delivery. What remains missing is a fine-grained analysis of the real drivers of actors’ behavior (in terms of the adopted instruments and consequent ability to impact reality) and the ways in which agents’ (e.g., healthcare providers’) misbehavior is prevented.

Policy instruments are the means by which governments “get things done” (3, 19), steer policies and improve the performance of existing policies. The relevance of policy instruments to every stage of policymaking has been recognized, as policy instruments represent one of the main research topics investigated by policy scholars (20–22). Furthermore, there is an increasing number of empirical studies on the effectiveness of policy instruments, especially in sectors such as innovation policy, environmental policy and climate change policy (23–27). This renewed attention to policy instruments has only marginally focused on the study of health policy (28), despite their relevance as the micro dimension of governance arrangements. In fact, only a few studies have focused on an instrumental perspective that is theoretically driven (29, 30), while a few more studies have offered a descriptive approach to instruments (31, 32). To be sure, increased attention has been devoted to policy instruments in healthcare studies due to the rise of studies on new public management in the field, as its application implies a prominent role of market or market-like systems and voluntary instruments of persuasion (33–35). However, the analysis of policy instruments is very often circumscribed, and attention is devoted either to specific policy instruments or to adoption of an instrumental perspective on specific healthcare subfields. For example, specific attention has been paid to instruments assumed to improve efficiency in health policy and policy instruments that directly target patients’ behavior, such as cost sharing, or physicians’ behaviors, such as provider payment methods (36, 37), to specific types of instruments for driving integrated care (38) and collaborative governance (39), to specific programs such as health promotion (30, 40), to tobacco control (41), and to health databases (42). It must be noted that, not unexpectedly, there is an enormous body of literature on nudges (43, 44).

However, a systematic comparative analysis on how institutional arrangements and governance principles are operationalized in policy outputs (and thus in concrete interventions) is clearly lacking. Thus, there is a deep gap in terms of understanding the micro dimension of health policy. This gap does not allow an in-depth understanding of the content of health policies and the extent to which current governance systems achieve the expected results.

## 3. A micro policy design perspective for comparative health policy

There has recently been growing interest in the development of an analytical framework focused on the content of policy design. There is a flourishing stream of research inspired by the institutional grammar proposal of Elinor Ostrom (45–49); there have also been interesting attempts to find hierarchies in design content (29), while there is increasing attention to whether and how micro changes in policy goals are connected with micro changes in policy tools (50) and how these connections also work in health policy (51). Finally, there are interesting conceptualizations that also pay attention to the dimension of implementation in the design of policy instruments, as in Schaffrin et al. (52), who propose six dimensions of calibration for climate policies (objectives, scope, integration, budget, implementation, and monitoring).

Cashore and Howlett (53) clarify the multi-level nature of the content of policies by distinguishing their aims and means at three different levels of abstraction: the *macro* level of general policy goals and the general principle of governance; the *meso* level, which refers to the venue in which policy objectives are implemented and the policy instruments chosen to implement the governance general principles; and the *micro* level, where policy goal targets and the method of delivery of instruments are chosen and designed. This method of conceptually disaggregating the content of policies allows us not only to better describe their internal complexity but also to show that the components of the micro level are those that impact reality and thus are the real drivers of policy outcomes. Applying this conceptual scheme to health policies makes it clear that to achieve systemic objectives (e.g., access, quality, equity, patient satisfaction, accountability and efficiency), it is necessary to set some programmatic goals (for example, competition, integration, decentralization, universal health coverage, and patient empowerment) that must then be translated into operational goals. At the same time, these high-level policy goals are coupled with instruments; that is, choices must be made between general guiding principles on instruments (state, market and/or collaborative governance) and translated to choices on the type of instruments to be adopted at the meso instrumental level and applied through the appropriate design at the micro level.

The meso level of instrumental choice is the one at which policymakers choose the type of instruments to be adopted. While there are different typologies of policy instruments that can be used to shed light on decision-making at this level (21), we develop our proposal by selecting four distinct families of key policy instruments that have different capacities to induce behaviors: *expenditure, regulation*, *information,* and *taxation* (19, 54, 55). Each family represents a specific mode of inducement. Expenditure involves remuneration, regulation involves behavioral control, information interventions leverage persuasion, and taxation—depending on how it is designed—can involve both behavioral control and remuneration. Notably, all four families of tools can feature different degrees of coercion in their application based on how freely individuals can choose alternatives. Taxation can be quite coercive when a general tax increase is established, but it can also be characterized by a low degree of coercion when many targeted tax exemptions exist. Regulation can be strong or weak depending on the type of behavioral prescriptions involved. Expenditure may be noncoercive in the case of subsidies, but it can also be very demanding in the delivery of targeted funding. Information interventions can be coercive when compulsory disclosure is imposed or less coercive when monitoring is applied. This means that in each of the four families of policy instruments, there are various specific types of instruments, which we define as “instrumental shapes,” through which regulation, expenditure, taxation and information can be specifically designed to achieve the expected policy outcomes. Table 1 offers several nonexhaustive examples of this conceptualization by listing some of the most relevant and widely used instrumental shapes in the health policy domain.

**Table 1:** Instrumental shapes in health policy (nonexhaustive examples).

Regulation	Expenditure	Taxation	Information
Public ownership and funding of health facilities Mandates to take out an insurance policy Access to specialist care: gatekeeping vs. direct access Authorization and accreditation of providers. Compulsory prerequisites to practice and/or receive public payment. Performance standards. Licensing/certification Compulsory guidelines Antitrust actions by competition authorities Regulation of insurers’ behavior. Open enrolment. Group−/community-rated premiums. Obligations to renew insurance policies Free choice of insurer (within mandatory schemes) Acknowledgement of patient rights Definition of a basic care package	Fee-for-service remuneration of providers Remuneration by capitation Remuneration of professionals by salary Pay-for-performance Extra billing Payment per day/*per diem* Case-based reimbursement [i.e., diagnosis-related groups (DRGs)] Global budgeting/block contracts Transfers to decentralized entities based on a reward system General practitioner (GP) budget holding	General and earmarked taxes. Salary-based contributions Compulsory health insurance premiums User charges Charges for not having health insurance Tax exemptions Tax incentives (to take out voluntary insurance) Tax subsidies for private health insurance	Quality assessment. Ranking and assessment of providers. Performance ratings Nonbinding accreditation Medical education/training of healthcare professionals Health promotion. Information and awareness campaigns Transparency. Data collection and analysis. Records. Statistics Nonbinding guidelines Promotion of best practices and benchmarking Patient empowerment Patient participation (open meetings, public workshops, forums, etc.) Patient experience/satisfaction surveys

However, for these instrumental shapes to be concretely applied to the real world of health policy, they must be delivered through a specific design package, which is the real determinant of the impact of the instrument itself. Thus, the micro level of policy design emerges as pivotal in analysing health policy. If we want to compare, for example, the characteristics of the role of public ownership in health to understand similarities and differences and to grasp whether and how this instrument has effects on the outcomes, then focusing only on the type of instrumental shape is not useful and can even be misleading. What is necessary, then, is to unpack the fundamental constitutive (and designed) characteristics of each instrumental shape.

According to Ingram and Schneider (56) and Salamon (3), any specific instrumental shape is a package of different elements characterizing the various aspects of public actions to induce behaviors in accordance with decision-makers’ expectations. Thus, while each instrument is expected to activate specific mechanisms with which to induce the expected behavior [compliance/obedience, remuneration/utility maximization or persuasion/changes in perceptions (19, 56)], the delivery component shows how instruments enforce their action on a target. Thus, it is the delivery package that concretely drives policy outcomes, based on the features of other adopted instruments and contextual factors.

**Figure 1:**
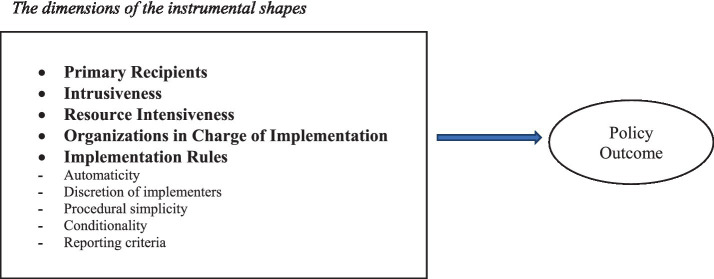
The micro dimension of policy design: the instrumental shapes.

In Figure 1, we propose selected dimensions along which the policy design content of instrumental shapes can be analysed.

These five dimensions are those that we consider the most relevant to the design of an instrumental shape in health policy (they can be changed based on theoretical assumptions or empirical evidence, but what is important here is to make the operating logic of the proposed framework clear).

- The **primary recipients** constitute the subjects to whom the measure is directly addressed and on whose activation the achievement of the objective depends. The primary recipients may coincide with the “ultimate recipients,” as in the case where individual physicians are remunerated on a fee-for-service basis for the volume of services actually provided (the incentive directly “hits” the actor whose behavior is to be influenced). The primary recipients may, however, not coincide with the “ultimate recipients.” One may, for example, incentivize the productivity of salaried doctors by rewarding or sanctioning the director of the local health agency. One could influence employees to cycle to work by giving a bonus to the respective company. In the latter two examples, the assumption is that the primary recipients (the director of the local health agency and the private company) will act to influence (in turn) the behavior of the “ultimate recipients,” i.e., the respective subordinates. There are five main possible types of primary recipients to which the instrumental shape can be tailored: (1) the entire population or certain subgroups of it (e.g., retired people, disabled people, young mothers); (2) public health organizations (such as local health authorities or public hospitals); (3) private organizations (e.g., insurance companies, private hospitals, pharmaceutical companies); (4) health professionals (doctors, nurses, etc.); and (5) subnational levels of government.
- **Intrusiveness** refers to how much the designed instrumental shape constrains the decision-making and behavioral autonomy of the actors involved. Sanctions are more intrusive than incentives, although the higher the incentive is, the greater the intrusiveness. Individual evaluations of doctors, for example, are less intrusive if they are not made public.
- **Resource intensiveness** regards the stock of organizational and financial resources that are designed to be involved in the implementation of the instrumental shape. This dimension may be absent in the design, meaning that when the instrumental shape is adopted, it is funded by existing organizational and financial resources, or could be accounted for with additional resources. For example, a vaccination campaign can be approved without an additional budget, or on the contrary, more resources can be allocated to recruit new staff, procure vaccines, set up new vaccination hubs, and upgrade logistical infrastructure.
- **Organizations in charge of implementation** are public or private organizations—with the exception of the primary recipients—that are assigned any role in the implementation of the instrumental shape. They can have at least five types of functions in the implementation of the intervention: delivering, coordinating, controlling, advising, and/or paying. This dimension is relevant for understanding the complexity of the implementation of the instrumental shape (the higher the number of administrative agencies and private actors involved, the more complicated and difficult the implementation will be).
- **Implementation rules** represent the procedural side of the instrumental shape. They can indicate:

- the extent to which the implementation of the instrumental shape is immediate or, on the contrary, requires further action (the level of **automaticity**);
- the amount of discretion left to the implementers in terms of adapting the instrumental shape to their related context (**implementers’ level of discretion**);
- the density of the procedures that must be followed to implement the instrumental shape (level of procedural **simplicity**);
- the extent to which provisions are triggered or change upon the occurrence of certain contextual conditions (level of **conditionality**). For example, certain restrictive measures come into force only when the number of confirmed cases of infection exceeds a certain threshold; and
- The extent to which monitoring and evaluation criteria and procedures are made explicit and detailed from the outset (**level of straightforwardness and detail of reporting criteria**).

## 4. Illustrative applications of the proposed analytical framework

To make the advantages of the proposed analytical framework clear, we apply it to examine the policy design of two specific interventions: maximum waiting time guarantees and vaccination mandates.

### 4.1. Maximum waiting time guarantees

As is widely known, long waiting lists are a point of discontent for many healthcare systems, especially in countries adopting the national health service (NHS) model (57–59). Among the various strategies that can be adopted to reduce waiting lists, several national governments decided to address the problem by setting maximum waiting time guarantees, under which citizens are entitled to receive prescribed medical treatment within a specified time frame (57, 60). One of the first countries to introduce this instrument was Sweden in 1992 (61). Following the Swedish example, many other countries have adopted the maximum waiting-time guarantee instrument, including Denmark, Norway, Finland, the Netherlands, New Zealand, Ireland, Italy, Portugal, and Spain (57, 59, 60, 62–64). These guarantees may appear—at first glance—to be a standard instrument applied in different countries in a uniform manner. However, this is not the case. This instrument can be designed in many different ways.

#### 4.1.1. Primary recipients

Maximum waiting time guarantees can be calibrated for different recipients (e.g., patients, hospital facilities, and territorial health agencies). The recipients may be all residents, with the maximum waiting time guarantee regarded as a patient’s right that any citizen can assert. Alternatively, the maximum waiting time can be conceived as a performance target to be achieved by hospital facilities. The actors who are “sanctioned” in the case of excessively long waits vary by country: they may be individual hospital facilities, local health agencies, subnational governments or the NHS as a whole. In some cases, financial rewards are envisaged for senior health administrators who ensure compliance with maximum waiting times (65).

#### 4.1.2. Intrusiveness

The maximum waiting time guarantee can be introduced with different degrees of intrusiveness. In many countries, including Sweden, Norway, and Denmark, this measure is introduced as a right of the patient and as an obligation on the part of public health facilities: if the maximum time limit is not respected, patients are allowed to use private providers free of charge; the cost of private providers falls on the public health agencies that have not respected the maximum time limit. Failure to comply with maximum waiting times therefore results in financial compensation for the patient and a penalty for the noncompliant public providers. Other countries—including England and Finland—have adopted a different version of the instrument, associating a financial incentive (or disincentive) with the waiting time targets: public hospitals that respect the maximum time limit receive extra funding, while hospitals not able to meet these targets are penalized. In this case, the degree of intrusiveness of the instrument depends largely on the size of the premium and the penalty. In some cases, for example, in England, the penalties can be severe: senior health administrators can lose their job if waiting time targets are not met (57). There are further countries where the maximum time guarantee has been introduced as a primarily informational instrument, with maximum waiting times considered a quality standard to aim for. In Italy, for example, local health authorities and regional health systems are required to monitor waiting times and make the results of this monitoring public on their website. However, noncompliance with these maximum times does not in practice lead to sanctions or incentives from the national government.

#### 4.1.3. Resource intensiveness

In some countries (including England, New Zealand, and Portugal in the early 2000s), the maximum waiting time strategy is supported by a dedicated budget to allow hospitals to make structural investments and increase their productivity. In other countries, the maximum waiting time strategy has not been accompanied by additional resources. The resources intended to support this instrument are not only financial but may also include the management of health and administrative personnel, computer systems for monitoring waiting lists, public communication tools, and more.

#### 4.1.4. Public and private organizations in charge of implementation

In the design of a policy instrument, great importance must be attached to the identification of public agencies and private organizations in charge of implementing the measure. Depending on the country, the implementation of the maximum waiting time guarantee may be the responsibility of the ministry of health, regional health authorities, local health agencies, or individual hospitals. Who should monitor waiting times? In many countries, waiting times are monitored by providers themselves; in other cases, external agencies are entrusted with this task (in Finland for instance, the monitoring of waiting times is entrusted to the National Supervisory Authority for Welfare and Health).

#### 4.1.5. Implementation rules

Finally, we come to the procedures to implement the policy instrument. A first procedural dimension concerns the level of *automaticity*. In some cases, the maximum waiting time guarantee has been made immediately operational by law (corresponding to a high level of *automaticity*). In other cases, once the principle has been established in general terms, its effective application has been postponed for implementation through further legislative measures.

A second procedural dimension concerns the level of *discretion of implementers*. In some countries, including Italy, maximum waiting times vary according to the urgency of the healthcare service. Individual doctors determine the degree of urgency of an examination, a specialist visit or a treatment. In this respect, doctors are given great discretion, and their decisions impact the calculation of waiting times.

A third dimension to be considered is *procedural simplicity*. In some countries, the procedure to comply with the maximum waiting time is simple and straightforward for the patient, whereas in other countries, this procedure is complicated and time consuming.

With regard to the *conditionality* dimension, penalties against hospitals that do not comply with maximum waiting times can come into force only when certain conditions are met. This is, for example, the case in the Emilia-Romagna region in Italy (65), where certain “disincentives” for hospitals, managers and doctors are applied only when the percentage of patients exceeding the maximum waiting times reaches a certain threshold.

A further procedural dimension is the characteristics of *reporting criteria*. In this regard, one element of great importance is the criteria used to estimate waiting times (59): they may be quantified retrospectively (once the patient has received the service) or as the expected waiting time (a forecast made at the time of diagnosis). National legislation often does not indicate in detail the criteria by which waiting times are to be calculated. This decision can be left to lower levels of government, to the agencies responsible for monitoring, and sometimes even to individual providers.

### 4.2. Vaccination mandates

It is commonly repeated that vaccinations are among the most effective, safest and cheapest public health interventions to prevent infectious diseases (66, 67). For this reason, many national governments share the goal of promoting mass vaccination campaigns. However, as the recent case of the COVID-19 vaccine has confirmed, in almost all countries, a not insignificant part of the population is reluctant to be vaccinated for a variety of reasons. To overcome this “vaccine inertia,” governments can use a variety of policy instruments that can be placed along a “ladder of intrusiveness” (31, 68–70) and that range from information tools (awareness campaigns, moral suasion) and material incentives and disincentives to personal restrictions (e.g., lockdowns for the unvaccinated). The imposition of vaccination mandates represents the top rung of this ladder of intrusiveness.

We aim to show that vaccination mandates are not a unitary instrument but can be designed “*in different shapes and sizes*” [(71), p. 7378].

#### 4.2.1. Primary recipients

Let us start with the recipients of the policy instruments. Depending on the type of vaccine, the vaccination mandate may concern the entire population or only certain target groups (72). Some mandates, for example, target only children, others only frail groups, and others only certain categories of workers. Consider the case of the COVID-19 vaccine. There are countries, including Indonesia, Ecuador and Austria, where the vaccination requirement has been extended to the entire adult population. In other countries, including France, Germany and Hungary, compulsory vaccination has been introduced only for certain categories of workers, e.g., healthcare workers, school staff, and the armed forces (73). In the United States, the vaccination requirement has affected federal employees. In Greece, the over-60 population has been obliged to be vaccinated.

#### 4.2.2. Intrusiveness

Compulsory vaccination is a regulatory instrument that is highly coercive in nature. Even with respect to this instrument, however, one can identify different nuances of intrusiveness, depending on the severity of the sanctions envisaged. Sanctions for noncompliance with vaccination mandates may be monetary or nonmonetary and may even go as far as imprisonment (71, 74). Many national and subnational governments, for example, in Australia, have introduced the “*no jab, no job*” rule: in such cases, unvaccinated workers are sanctioned with the suspension of the employment relationship (without revenue). In relation to some childhood vaccines, in several countries, school enrolment is conditional on presentation of a vaccination certificate (74), with unvaccinated children excluded from schools, kindergartens, and day care centers. For those who are not vaccinated, fines may apply. It is evident that harsher sanctions are perceived as more “intrusive.” It is interesting, in this regard, to compare the penalties formally provided for in some European countries that have made vaccination against COVID-19 compulsory: in Italy, for those over 50 who violate the vaccination mandate, a one-off fine of 100 euros has been stipulated; in Greece, the fine is 100 euros per month; in Austria, the stipulated fines are 600 euros every 3 months, up to a maximum of 3,600 euros (73).

#### 4.2.3. Resource intensiveness

Governments introducing a vaccination mandate can either allocate extra funding dedicated to implementing the measure or introduce the mandate on a “zero budget” basis. For example, the 2017 Italian reform that introduced 10 compulsory vaccinations for children explicitly provided for the allocation of an *ad hoc* budget to be used for communication campaigns and possible monetary compensation for vaccine-caused damages (75). Many of the governments that have introduced some kind of compulsory COVID-19 vaccination requirement have not allocated a “dedicated” budget. However, it should be noted that the resources involved are not necessarily only financial. Most governments that have made the COVID-19 vaccine compulsory (for the whole population or even just a part of it) came to the decision to do so only when they had accumulated large stocks of vaccine doses and when it had been demonstrated that the “organizational machine” for administering the injections could vaccinate a high number of patients every day.

#### 4.2.4. Public and private organizations in charge of implementation

When vaccination mandate is introduced, it is necessary to ascertain (1) who is in charge of administering the jabs, (2) who controls who has been vaccinated and who has not, and (3) who is in charge of enforcing sanctions and collecting fines. Usually, the entities in charge of these three tasks are separate public or private agencies that have to coordinate with each other, cross-referencing the data at their disposal. Vaccines are usually administered by territorial health agencies. However, vaccines can also be administered in other locations: in hospitals (public and private), in hubs set up in municipalities, in family doctors’ surgeries, in workplaces, etc. Who monitors compliance with vaccination mandates? In the case of the COVID-19 vaccine, this task has often been assigned to local health authorities. However, this burden can also be placed on municipalities. In some countries, control is delegated to employers (which may be public but also private). In the case of childhood vaccinations, control is often entrusted to school facilities. The enforcement of sanctions may be assigned to a variety of public entities: the ministry of health, municipalities, local health authorities, the revenue agency, etc.

#### 4.2.5. Implementation rules

Finally, let us consider the implementation procedures by which the vaccination mandate is introduced. A first procedural dimension concerns *automaticity* and the possible existence of a time limit on the obligation. In Austria, for example, a vaccination mandate for the entire over-18 population was introduced on 5 February 2022. However, this obligation was not immediately implemented: for the mandate to take effect, the opinion of an expert committee was needed. This committee of experts decided to postpone the entry into force of the obligation. At the beginning of March 2022, the Austrian government decided to follow the opinion of these experts, postponing the application of the vaccine mandate (73).

A relevant dimension of compulsory vaccination concerns the categories of persons exempt from the requirement. It is well known that vaccines can be dangerous for people suffering from certain diseases and are therefore not recommended. In this regard, legislation may allow doctors more or less discretion. In many countries, including Italy, the decision on exemption from compulsory vaccination for ‘health reasons’ is left to individual family doctors (each of whom may apply different criteria). In other countries, in order to obtain an exemption, patients are required to present the results of specific medical examinations. In the latter case, the *discretion of implementers* is clearly less.

In some countries, one may be exempted from vaccination not only for medical reasons but also for ethical or religious reasons. The issue of different types of exemption from compulsory vaccination is also linked to the dimension of *procedural simplicity*: what procedures are in place to prove that one meets the requirements for a formal exemption? In some countries, the patient does not have to do anything because the health authorities already have the necessary information in their databases to proceed with the exemption. In other countries, citizens must instead take action by delivering a medical certificate to health authorities. To prove exemption on ethical or religious grounds, sometimes a self-declaration is sufficient; in other cases, the procedure to be followed is more complicated (71, 74).

Regarding *conditionality*, the vaccination obligation can only be implemented if certain conditions are met. The example of the vaccination mandate in Austria (conditional on the opinion of an expert committee) falls into this category. However, there may be other examples where the implementation of the mandate is linked to the vaccination coverage rate: if the coverage is below a certain threshold (e.g., the herd immunity threshold), the obligation enters into force but is suspended as soon as the vaccination coverage rate exceeds the threshold.

Regarding the *reporting criteria*, once a vaccination mandate has been introduced, it is essential to define precisely who has complied with the obligation and who, on the contrary, is noncompliant. This has important implications for how the vaccination campaign is monitored and evaluated. Many vaccinations (e.g., vaccinations against Covid-19 or most childhood vaccinations) involve several doses administered at intervals. In some countries, those who have started a vaccination cycle are counted as having complied with the obligation, whereas in others, only those who have completed the vaccination cycle are counted. For the purposes of monitoring, should those who are immune because they have already contracted the disease be counted as having been vaccinated? How should those who are exempt due to disease or for ethical or religious reasons be counted in monitoring and assessment?

## 5. Discussion: health policies under the microscope

When comparing health systems and health policies in different countries, one runs the risk of considering individual policy instruments to be standard measures. In many countries, for example, hospitals are remunerated according to a DRG system, but DRG-like systems are by no means equal. Although they share a common approach, they can be designed differently, thus providing varying incentives depending on how the remuneration system is organized. Additionally, in many countries, the government provides tax incentives for citizens who voluntarily purchase a private health insurance policy. However, the way these incentives are shaped (who the recipients are, what types of policies are incentivized, the amount of the incentives, how the incentives are applied, etc.) can make a great difference.

A limitation of many comparative health policy studies is therefore that they consider policy instruments to be similar when they are not. This limitation stems from the fact that the analysis of policy instruments often remains at a general level rather than going into depth. What we propose in this article is therefore a radical change of perspective that leads to analysing individual policy instruments in greater detail by breaking them down into their different components.

To use a metaphor, we propose to study health policies (and their instruments) by putting them under a microscope. Only in this way is it possible to focus on and identify the different components of policy instruments, and to fully grasp the similarities and differences between the multiple strategies with which the instruments are shaped.

First, the focus on micro dimensions allows us to grasp the real content of policy designs in health policy. This perspective, then, suggests that health policies should be compared not only in terms of the types of instruments adopted (at the risk of being trapped in dichotomies such as more or less regulation and more or less market-oriented instruments) but also in terms of the micro design of different policy interventions and policy programs. In this way, comparative analysis can better illuminate what health policy truly is, how it works and how healthcare is concretely delivered.

Second, the proposed micro perspective, thanks to its ability to account for the details of instrumental design, can be very useful to better clarify what is truly at stake in reforming health policy and thus can help to explain the political dynamics of the related policy-making processes. What is truly at stake in health policy-making is not only general principles of governance or general goals (equity, access, quality, etc.) but also the way in which these goals are pursued. In addition, the ‘How we do this?’ question is politically highly relevant because it implies not only a normative dimension (which could relate to preferences for more or less intrusiveness, for one primary recipient over another one, or for more or less automaticity) but also the involvement of concrete interests of stakeholders (for example, insurance companies would certainly prefer a low level of intrusiveness in policy design).

Third, focusing on the micro dimension of policy design and thus on a more granular and in-depth analysis of the different components of the design of the instrumental package makes it possible to assess how effective the instrument is and under what conditions it is effective. This is a very relevant point when the issue of policy outcomes is under investigation. All in all, what, *ceteris paribus*, can activate the expected process leading to the planned policy outcome is not the type of health system in which the policy intervention is introduced but rather the exact policy instruments or set of instruments adopted. While we are perfectly aware that policy design must be contextualized and that environmental and contingent conditions could matter for performance and, overall, for the choice of policy instruments and the characteristics of policy design and implementation, at the same time, the characteristics of the instrumental delivery packages cannot be considered completely irrelevant. If designed properly, according to the context, they can actually contribute to driving the expected policy performance.

## 6. Conclusion and suggestions for further research

This paper offers a new framework committed to filling an analytical gap in comparative policy research—namely, the lack of systematic analysis of the content of policy designs in terms of the characteristics of the adopted instruments. We consider this framework a powerful tool to disaggregate the content of policy design (in terms of policy outputs) and to help assess the driver of policy performance in the sector from a comparative perspective.

Obviously, for the significance and reliability of this framework to be proved, more than the two applications proposed above are required. The framework needs to be applied to a longitudinal comparative reconstruction of policy interventions at least for a specific policy program. This empirical application will show whether and how the dimensions proposed to grasp the characteristics of micro policy design are suitable and whether and how they should be partially modified and/or reconceptualized.

At the same time, our effort opens the door to reconceptualizing some relevant themes in comparative health policy and thus suggests avenues for further research. At least three streams of research can be proposed here (which can also be considered also independently from systematic research that applies the framework itself).

First, there is the question of the real coherence between the macro and meso levels of governance arrangements and the micro level. Are most instrumental packages designed according to the general prevailing governance principles, or could there be a significant level of disconnection? If this is the case, then this disconnection becomes relevant and needs to be explained.

Second, there is the issue of policy change. If, as assumed by the framework, the micro dimension matters a great deal in terms of defining the content of policy design and of its effects, then there is no guarantee that a profound change in either governance principles or the adopted policy instruments (e.g., a shift from a state-centered type of intervention to a more market-oriented policy or from payment per day to payment per case for hospitals) will modify the effectiveness of the services provided to the public (if the micro design is not appropriate). Thus, there is the issue of better focusing on how micro design contributes to policy change in terms of changes in policy performance. In fact, it could be the case that radical changes (in the macro and meso components of policy design) may deliver poor performance while small incremental changes (usually those to micro dimensions of policy design) can bring about significant positive change.

Finally, there is the issue of how micro designs impact reality in terms of activating the processes and behaviors that lead to the expected performance. Are policy instruments the causes of policy performance? Are they activators of specific mechanistic chains?

Indeed, focusing on the granular dimension of policy design can drive the exploration of new ways to understand and explain healthcare policies and thus also to improve them.

## Data availability statement

The original contributions presented in the study are included in the article/supplementary material, further inquiries can be directed to the corresponding author.

## Author contributions

All authors listed have made a substantial, direct, and intellectual contribution to the work and approved it for publication.

## Funding

This work was supported by the Ministry of Education - Italy - Project PRIN 2017 Who Advises What, When and How? Policy analysis capacity and its impact on Italian policy-making.

## Conflict of interest

The authors declare that the research was conducted in the absence of any commercial or financial relationships that could be construed as a potential conflict of interest.

## Publisher’s note

All claims expressed in this article are solely those of the authors and do not necessarily represent those of their affiliated organizations, or those of the publisher, the editors and the reviewers. Any product that may be evaluated in this article, or claim that may be made by its manufacturer, is not guaranteed or endorsed by the publisher.
